# Elderly women’s experiences of self-sampling for HPV testing

**DOI:** 10.1186/s12885-020-06977-0

**Published:** 2020-05-26

**Authors:** Ruth S. Hermansson, Matts Olovsson, Catharina Gustavsson, Annika Kristina Lindström

**Affiliations:** 1 Department of Women’s and Children’s Health, Uppsala University, Uppsala, Sweden; 2 Clinical Research Center, Faculty of Medicine and Health, Örebro University, Örebro, Sweden; 3 Center for Clinical Research Dalarna, Uppsala University, Falun, Sweden; 4 School of Education, Health and Social Studies, Dalarna University, Falun, Sweden; 5 Department of Public Health and Caring Sciences, Family Medicine and Preventive Medicine, Uppsala University, Uppsala, Sweden

**Keywords:** HPV, Self-sampling, Cervical cancer, Prevention, Elderly, Women

## Abstract

**Background:**

Self-sampling for HPV testing, as an alternative to the conventional speculum based sampling, is highly acceptable to women of screening ages. The aim of this study was to describe older women’s (60 to 75 years) experiences of self-sampling.

**Methods:**

In Sweden a descriptive study with quantitative and qualitative methods was designed to collect data from a survey of women who participated in self-sampling for HPV testing. Individual interviews were done with women who tested positive in the first self-sampling, and were either negative in their second HPV test or were positive in their second HPV test, but without precancerous lesions or cancer.

**Results:**

Of 893 eligible women, 868 (97.2%) answered the survey. Among the surveyed women, 49.2% reported it was very easy to perform self-sampling, 46.8% answered it was easy and 2.0% answered it was not easy. A majority (58.9%) answered that they prefer self-sampling, 16.5% that they prefer sample collection by a healthcare provider, 23.7% did not have any preference and 0.9% did not answer the question. In the interviews, 13 of 16 invited women participated. Most of them reported that they prefer self-sampling because it was easy to perform, less embarrassing and less time consuming than a visit to a clinic. The majority of women reported that they were not worried when informed about having an HPV positive test. Overall, participating women with better knowledge about the significance of an HPV infection were more worried about having a positive HPV test.

**Conclusion:**

Cervical cancer remains a highly preventable disease through screening and early treatment. Our results indicated that vaginal self-sampling for HPV testing was a well-accepted method for cervical cancer prevention in this group of older women.

**Trial registration:**

https://www.researchweb.org/is/en/fouckfuu/project/272587. Registered 24 June 2019-retrospectively registered. www.researchweb.org

## Background

In Sweden, about 30% of cervical cancer (CC) cases occur in women older than 60, and the mortality rate is about 70% in this age group [1]. Cervical cancer in women above the age of 65 is usually discovered at advanced stages and the prognosis is poor [2]. During the past century, the average life expectancy has increased globally and in Sweden. The life expectancy for women is 84 years and many women over 65 are healthy, continue to work and have an active sex-life [3]. Since 2015, the Swedish Board of Health and Welfare have recommended sampling for cytology, every third year for women aged 23–29, sampling for HPV-testing every third year for women aged 30–49, and a supplemental analysis also for cytology for women who are about 41 years old. For women aged 50–64, sampling for HPV-testing is recommended every 7th year [4]. In Sweden most screening samples are collected by midwives and in the case of abnormality the patient is referred to a gynecologist.

The causal role of human papilloma virus (HPV) in the development of CC has been firmly established [5]. It is recognized that co-factors to a persistent HPV infection increase the risk for the development of invasive cancer. Cigarette smoking has been established as an independent risk factor for CC [6–8]. Other contributing factors are long term use of oral contraceptives, high parity, hormonal changes in the vagina, the inadequate function of the immune system and genetic instability [5]. HPV is sexually transmitted and most of the infections have no signs or symptoms. Indeed the majority of HPV infections, about 80%, clear spontaneously within 1 year after acquisition in young women but whether this occurs on the same extent in elderly women is unknown [9].

HPV 16 and 18 are responsible for about 70% of all cancers of the cervix and the vagina [5, 10]. The clinical value of HPV testing is well established, and HPV testing demonstrates superior efficacy for reducing the incidence of cervical cancer compared to cervical cytology [11]. Vaginal self-sampling is an appropriate option for HPV testing, since the results are fully comparable with those from samples collected by a healthcare provider, as a reliable method for HPV testing for women of screening ages [12, 13]. Repeat testing for HPV can be used to increase the specificity in the screening for CC [14, 15]. Cervical cancer is a highly preventable disease, and each new case can be seen as a failure. Cervical cancer screening programs in many countries stop at around the age of 65. There is however no clear evidence on what is the appropriate age to stop screening [16]. Curves that show age-specific incidence of CC in areas with established cervical screening programs have two peaks: one around 40 years and the other at around 75 years. In Sweden, the second peak has decreased only modestly with time [17].

Few studies have investigated HPV testing in older women, who are no longer included in the screening program [18, 19]. A recent study showed a high positive predictive value (PPV) for detecting high-grade squamous intraepithelial lesion (HSIL), by repeat HPV self-sampling and biopsies for histological analysis [15]. It has also been shown that the HPV test shows much higher sensitivity than cytology in detecting cervical dysplasia [18, 20]. Women’s attitudes to, and acceptability of, self-sampling has been evaluated in several studies showing that self-sampling was highly acceptable as an alternative to the conventional speculum based sampling [15, 16]. There are however no studies on acceptability of self-sampling for HPV testing in older women. The aim of this study therefore was to explore how older women (60 to 75 years) experience self-sampling at home.

## Methods

This is a descriptive study using quantitative and qualitative methods.

### Procedure and data collection in the survey

In a previous study 1500 women were randomly selected from the Swedish population register, with 375 women in each of the four age groups 60, 65, 70 and 75. These women were invited to perform self-sampling for HPV testing [15]. The participation rate was as follows, 62.9% (236/375) at age 60, 63.5% (238/375) at age 65, 59.5% (223/375) at age 70 and 52.3% (196/375) at age 75. The women received detailed written information about HPV infection, instructions for sampling and the opportunity to call for more information. In brief, 893 women performed self-sampling at home and each sample was returned in a prepaid postal envelope to the laboratory for HPV analysis. For full description of the self-sampling instructions see supplementary material (Fig. 1). Women with a positive first HPV test were sent a new self-sampling kit 4 months after the first test was done. All 893 who provided a self-collected sample for HPV analysis between autumn 2014 and spring 2015, were eligible for this study and sent a survey by regular mail. The questionnaire included 20 closed-ended questions concerning various aspects of the women’s health, early gynecological disease, lifestyle and two specific questions about their concerns regarding self-sampling. This questionnaire was constructed by the research group and sent by regular mail to the women. In the present study, only the questions about self-sampling were analyzed. They were asked:

1. How easy or difficult it had been to collect the sample at home:

a. very easy b. easy c. not easy.

2. Do you prefer self-sampling as compared to a sample collected by a healthcare provider?

a. yes b. no c. uncertain.

The survey also included an open-ended question about their opinions, experiences or advice on how the self-sampling could be improved.

### Procedure and data collection at interviews

Women were informed from the beginning, in writing, that if the HPV test was negative they would be considered at very low risk for cervical cancer and that there would be no further follow up. All sixteen women who tested positive in the first self-sampling, and either were negative in their second HPV test (n = 11), or were positive in their second HPV test but without precancerous lesions (n = 5), were invited to the interviews. These women were selected since they may reasonably have pondered upon the procedure and the test results to a greater extent than women with a negative test with no follow up. At the time of the study there was no follow up guidelines for older women with an HPV positive test without dysplasia. Thirteen of the 16 invited women agreed to participate and gave written informed consent.

Individual interviews were undertaken during the winter 2017–2018 according to a semi-structured interview protocol. The protocol contained open-ended questions designed to respond to the research questions of the study. The interviews lasted 20–30 min and were carried out by the first author (a female gynecologist). The interview was conducted in a place chosen by the participants (at home for example), where only the participant and the interviewer were present and undisturbed by others. The interviews were audio-recorded and transcribed verbatim by a research assistant.

The research questions investigated by the interviews were:

- women’s experiences about the pros and cons of performing self-sampling at home as compared to sampling by a healthcare provider.
- women’s knowledge about HPV infection and the relationship between HPV infection and cervical cancer.
- women’s experiences concerning notice of a positive HPV test and what feelings such information brought.

### Data analyses

The survey data were analyzed with SPSS (Statistical Package for Social Sciences) version 24. Fisher’s exact test was used. The interview data were analyzed using qualitative content analysis and undertaken in two steps [15]. First, the text files of the interviews were read as soon as they had been transcribed, in order to gain an overview of the material. Then, three of the authors (RSH; AKL; CG) performed the data analysis with a deductive approach by reading the text again and identifying meaning units, i.e. specific units of text consisting of a single word, a few words, or a few sentences relating to the research questions for the interviews [21]. Meaning units were condensed and coded, i.e. by labelling the content of the condensed meaning unit. Codes were then discussed and grouped together into predefined categories corresponding to the research questions investigated by the interviews. All authors participated in discussing and revising the interpretation until a consensus was arrived at.

## Results

### Survey

Of 893 eligible women, 868 (97.2%) answered the survey. The participation rate in each age group was 96.2% (60 years), 97.9% (65 years), 97.8% (70 years) and 96.9% (75 years) respectively. The background characteristics of the participants are shown in Table 1.

**Table 1:** Background characteristics of participants (n = 868)

Age, years (range)	60–75
HPV positive, n (%)	36 (4.2)
HPV negative, n (%)	832 (95.8)
Nulliparous, n (%)	66 (7.6)
Parity (1–7), n (%)	799 (92.4)
Never smokers, n (%)	757 (87.9)
Current smokers, n (%)	85 (9.8)
Previous smokers, n (%)	19 (2.2)
Single, n (%)	203 (23.4)
Having a partner, n (%)	664 (76.6)
Sexually active, n (%)	380 (43.8)
Not sexually active, n (%)	453 (52.2)
Sexual activity not reported, n (%)	34 (4.0)

The survey contained two specific questions about women’s experiences of self-sampling. To the question regarding how easy or difficult it had been to collect the sample at home, 49.2% answered very easy, 46.8% answered easy, 2.0% answered not easy, and 2.0% of the women did not answer this question (Table 2). In the same table, there are also details for each age group.

**Table 2:** Answers by age group on how easy or difficult it had been to perform self-sampling

Age group years (n)	Very easy n (%)		Easy n (%)		Not easy n (%)		No answer n (%)	
60 (227)	121	(53.3)	101	(44.5)	2	(0.9)	3	(1.3)
65 (233)	124	(53.2)	103	(44.2)	4	(1.7)	2	(0.9)
70 (218)	105	(48.2)	100	(45.9)	6	(2.8)	7	(3.2)
75 (190)	77	(40.5)	102	(53.7)	6	(3.2)	5	(2.6)
**Total (868)**	**427**	**(49.2)**	**406**	**(46.8)**	**18**	**(2.0)**	**17**	**(2.0)**

*There were no significant differences in the answers between age groups (p = 0.104)

To the question about the preference of self-sampling or sampling by a healthcare provider. Self-sampling was preferred by 58.9% while 16.5% prefer to have a sample collected by a healthcare provider, 23.7% did not have any preference and 0.9% of the women did not answer this question (Table 3). In the same table, there are also details for each age group.

**Table 3:** Answers by age group about sampling preference

Age group years (n)	Prefer self-sampling n (%)		Prefer health- care provider n (%)		Uncertain n (%)		No answer n (%)	
60 (227)	124	(54.6)	41	(18.0)	61	(26.9)	1	(0.4)
65 (233)	142	(60.9)	34	(14.6)	56	(24.0)	1	(0.4)
70 (218)	118	(54.1)	43	(19.7)	55	(25.2)	2	(0.9)
75 (190)	127	(66.8)	25	(13.2)	34	(17.9)	4	(2.1)
**Total (868)**	**511**	**(58.9)**	**143**	**(16.5)**	**206**	**(23.7)**	**8**	**(0.9)**

*There were no significant differences in the answers between age groups (p = 0.085)

In the survey, there was an opportunity for the participants to leave comments or an opinion on self-sampling. There were comments from 176 women, 75 confirmed that self-sampling was easy and uncomplicated, 26 women reported that they felt uncertain as to whether the sampling was performed correctly, 11 women reported that the brush was hard and uncomfortable and two of those women reported a little bleeding after sampling.

### Interviews

Thirteen women aged 60 to 75 years participated in the interviews. The results from the content analysis of the interviews pertaining to each of the three categories corresponding to the research questions, are summarized in Table 4 and appear in further detail below, with quotations in text of a different font.

**Table 4:** Categories and examples of codes and meaning units in each category

Categories	Codes in each category	Examples of meaning units
HPV self-sampling compared with sampling by a healthcare provider.	- Easy to perform self-sampling at home - Easy to understand the instructions - Preference of self-sampling compared with sampling by a healthcare professional at a clinic.	- *It was very good, I mean, that you could do it at home and then just send it.* *-Very easy, it was great!* *- It was actually very nice to do it myself instead of lying down in this chair*
Knowledge and concerns about the relationship between HPV infection, and risk for CC.	- Low knowledge about HPV - Low knowledge about the relationship between HPV infection and CC - Low knowledge about CC screening and prevention	- *Now I know a little more after reading the information, but before I didn’t know so much* *“Yes... to start with I didn’t actually know anything … but later I read a bit that it can cause cancer …*” *-No, I do not know so much. But I know there is a vaccine for little girls.*
Experiences and feelings about an HPV positive result.	- Worries regarding an HPV positive result - No feelings of shame about having an HPV positive result - Need for more information	*-I was not worried! A little surprised, maybe.* *-I was a little frightened. I didn’t know much about it. I wondered if I had cancer.* *-I did not think so much about this! I have been married to the same man for more than 30 years.* *-It felt safe to know that you could call for more information if you want!*

*CC* Cervical cancer.

### Self-sampling at home compared with sampling by a healthcare provider

Most of the women interviewed responded that it was relatively easy to take a vaginal self-sample. One of the women had difficulties performing self-sampling due to rheumatic disease. Only one woman reported feeling unsafe and troubled with self-sampling and preferred sampling by a healthcare provider. The majority of the women interviewed experienced that self-sampling was practical, less embarrassing and less uncomfortable than sampling by a healthcare provider. A very important factor was that the self-sampling was less time consuming than visiting a clinic. Most of the women preferred self-sampling due to the difficulties related to obtaining an appointment at the healthcare center.

“If it is just as good then it is great to do it at home, so that I don’t have to take up someone else’s time”.

“I’m not afraid to test new things. And also, I believe that it’s effective for me because I don’t have the time, so to speak. I mean, having to go somewhere, sit in line and wait and then to get back home... and in addition, I don’t have a car anymore.”

Almost all of the women interviewed were in favor of self- sampling instead of a vaginal examination with instruments performed by a healthcare professional. The majority of the women felt safe and comfortable with the information and instructions that they received with the study invitation and would prefer self-sampling if available.

“It was better than having a male doctor and having to lie down with spread legs”.

### Knowledge and concerns about HPV infection and the relationship between HPV infection and the risk of cancer disease

Most of the women interviewed reported limited knowledge about HPV and the relationship between HPV infection and CC. More than half of the women knew, or at least had some vague idea, that HPV is a sexually transmitted infection. About half of the women reported that they knew that for as long as they were sexually active there was a reason to participate in this study.

“I have read something … .my mother died because of cervical cancer and I want to prevent it...”

Most of the women did know about the HPV vaccine and its use for cervical cancer prevention.

“I know that HPV can cause cancer. I don’t know how exactly, but I know that young girls can get an HPV vaccination before their sexual debut”.

Not one of the women interviewed knew the reason why the screening program ends at the age of 60. Some of the women thought that it was age-related discrimination and a few assumed that it was for economic reasons.

“They do not care about us older women!”

“No, I don’t understand … I mean you have sexual intercourse after 60 as well …”.

Many of the women were confused about whether HPV resolves on its own and whether a treatment exists. There was also confusion about the correlation between the woman’s age and the risk of CC. Some of the women believed that after 60 years of age the risk of CC disappears. The interviewed women did not know that there are many different types of HPV. Few of the women knew that HPV could cause other types of cancer besides cervical cancer.

A few of the women reported that they were worried at first; about whether they could be confident about having performed the self-sampling correctly.

“I was worried! Did I do it right?”

Not one of the women interviewed reported being worried about the accuracy of the test or other factors in the procedure, such as sending the test by mail. All of the women felt comfortable knowing that making contact with the gynecologist responsible for the study, by either phone or e-mail, was possible.

### Experiences and feelings about an HPV-positive result

All of the women interviewed had tested positive for HPV on the first test. Most of the women reported that this information did not cause anxiety. The majority of them expressed that they had faith in the healthcare service and that they did not feel worried while waiting for the result of the second HPV test.

“I was not afraid! I thought that a new test would be done and if necessary, I will receive help from healthcare services”.

A few of the women were afraid of the potential implications of an HPV infection. For example, one woman was worried about her husband and the possibility that he was also at risk of having cancer. Another woman was worried about infecting someone else with HPV. Only one woman was very afraid concerning the risk for CC, that is, afraid that the HPV infection had already turned into a cancer disease.

Overall, those women interviewed who has more knowledge about HPV had expressed more worries about having a positive HPV test.

“I thought like: do I have a ticking bomb? Could this become cancer?”

More than half of the women interviewed were in a stable intimate relationship and no one reported that a positive HPV test had affected the relationship adversely. One women had had a short-term relationship, which was the principal reason for her taking part in the study. Not one of the women interviewed reported feeling shame or anxiety concerning the notice of a positive HPV test.

## Discussion

This is the first study focusing on older women performing self-sampling at home for the analysis of HPV. The participants in this study represent Swedish women too old for the national CC screening program, which at the time of the study ended at 60 years of age. The aim of this study was to describe the experiences of elderly women performing self-sampling for HPV testing.

We found high acceptability for self-sampling, which is consistent with previous studies on younger women [22–24]. Among surveyed women, the vast majority responded that it was easy to take a self-sample. Only 2% of the participants in the survey indicated that self-sampling was not easy. To the question about the preference of self-sampling compared to sampling by a healthcare professional, more than half of the women reported that they prefer self-sampling, which is also in agreement with previous studies [24, 25]. Nelson et al. reported in a review that self-sampling for HPV testing is generally well accepted by women not attending the screening program, and is preferred to clinician-based sampling [22]. There was no difference in acceptability between the age groups in this study.

The interviews aimed to capture women’s experiences and concerns about self-sampling, their knowledge about HPV and their experiences concerning the notice of a positive HPV test. The women interviewed had almost no knowledge about HPV testing and no one was familiar with self-sampling before participation in the current study. The participants in the interviews generally had good experiences of self-sampling at home. They could see advantages such as it being easy, comfortable, maintaining privacy, and it was also less time and resource consuming than an appointment at a clinic. These results are surprisingly similar to what has been shown in earlier studies performed on younger women [23, 26]. Most of the women in our study were confident with the self-sampling and the accuracy of the test result. Nobody was worried about using regular mail to send the sample and all seemed to be confident with the analysis. It is concluded that the elderly women in our study prefer self-sampling for HPV testing as a part of a cervical cancer prevention program for elderly women. Similar results were shown in a Finnish study conducted on non-attendees who performed self-sampling, where more than 80% felt confident with the self-sampling and a similar proportion trusted the test results [26].

A lack of knowledge about the relationship between the HPV infection and CC development, or the underestimation of the risk for CC, was found, and this could be one reason for the low level of worry and concern about a positive HPV test. The interviews showed that women with better knowledge about HPV were more worried about being HPV positive.

Most of the interviewed participants had a stable relationship. We found that no one reported feelings of shame or anxiety on being diagnosed with HPV, and no one described that this knowledge had a negative impact on their relationship. These findings are not in agreement with other studies where they found that women were anxious about being HPV positive, since an HPV infection is a sexually transmitted disease that could have a negative impact on their relationship. A study conducted in London, on Hindu women, reported that the knowledge of a positive HPV test and the fact that no treatment exists, was a cause of concern and anxiety in those women, and they considered that this knowledge could have negative effects on their relationships [25]. O’Connor et al. reported that the shame and embarrassment expressed by some of the women interviewed, resulted from HPV being sexually transmitted. A few women feared they would experience stigma, and be judged promiscuous by their peers because of an HPV positive test [26]. It remains unclear why the notice of an HPV positive test does not cause feelings of shame or anxiety in the participants in our study. It might be that the women in the current study are much older and that many of them had limited knowledge about HPV.

In our study, the majority was comfortable and satisfied with the information and instructions that they received in the study invitation. This outcome has several practical implications, for example, including adequate and balanced information about HPV and the significance of an HPV infection, to prepare the woman for the coming test result and possible further examination.

We also found a high demand for, and intention to use, self-sampling in the future. The women in our study expressed that they would use self-sampling if it was available and most of them asked for the next time-point for sampling. Our findings are important because in Sweden life expectancy for women is high and about one third of the new CC cases occur in women above the age of 60 [1]. Self-sampling thus constitutes a superior alternative for also providing screening to elderly women. Moreover self-sampling has the potential to further reduce costs as it eliminates the need for an initial clinical encounter in the screening process [27, 28].

The strength of the study is the high response rate, with most women in each age group answering the survey, also that 13 of the 16 women eligible for the interviews gave written consent and participated in the interviews. A limitation is that the study was performed only in one region of Sweden. A larger number of participants may have resulted in more information. Another limitation might be that the women in the study were no longer included in the national CC screening program, which could have influenced their attitude to self-sampling as an opportunity to be screened.

## Conclusion

Our results indicate that vaginal self-sampling for HPV testing is a well-accepted method for cervical cancer prevention in this group of elderly women.

## Supplementary information

**Additional file 1.** Fig. 1 Self-sampling instructions.

## Data Availability

Data are available on request for any interested researcher to allow replication of results, provided all ethical and legal requirements are met according to GDPR. The General Data Protection Regulation for the European Union. The datasets generated and analyzed during the current study are not publicly available due to the modest size of the sample in the interview, and to avoid the participants being identified. The survey is only in Swedish and the interview questions are translated to English and submitted as supplement. Contact person, Center for Clinical Research, Dalarna, Uppsala University, Karin.bjorlin@regiondalarna.se Nissers väg 3, SE-79182 Sweden.

## Abbreviations

HPV: Human Papilloma Virus
CC: Cervical Cancer
PPV: Positive Predictive Value
HSIL: High-grade Squamous Intraepithelial Lesion
RSH: Ruth Sanchez Hermansson
AKL: Annika Kristina Lindström
CG: Catharina Gustavsson

## References

[1] Danckert B F, Engholm G, Hansen HL, Johannesen TB, Khan S, Kötlum JE, Ólafsdottir E, Schmidt LKH, Virtanen A and Storm HH. NORDCAN: Cancer incidence, mortality, Prevalence and Survival in the Nordic Countries Version 8.2. 2019.

[2] Darlin L, Borgfeldt C, Widen E, Kannisto P (2014). Elderly women above screening age diagnosed with cervical cancer have a worse prognosis. Anticancer Res.

[3] Beckman N, Waern M, Gustafson D, Skoog I (2008). Secular trends in self reported sexual activity and satisfaction in Swedish 70 year olds: cross sectional survey of four populations, 1971-2001. BMJ..

[4] Socialstyrelsen LsmcoH-t (2015). Livmoderhalscancer – screening med cytologi och HPV-test - Socialstyrelsen.

[5] Bosch FX, Lorincz A, Munoz N, Meijer CJ, Shah KV (2002). The causal relation between human papillomavirus and cervical cancer. J Clin Pathol.

[6] Fonseca-Moutinho JA (2011). Smoking and cervical cancer. ISRN Obstet Gynecol.

[7] Huttunen R, Laine J, Lumio J, Vuento R, Syrjanen J (2007). Obesity and smoking are factors associated with poor prognosis in patients with bacteraemia. BMC Infect Dis.

[8] Appleby P, Beral V, Berrington de Gonzalez A, Colin D, Franceschi S, International Collaboration of Epidemiological Studies of Cervical C (2006). Carcinoma of the cervix and tobacco smoking: collaborative reanalysis of individual data on 13,541 women with carcinoma of the cervix and 23,017 women without carcinoma of the cervix from 23 epidemiological studies. Int J Cancer.

[9] Woodman CB, Collins S, Winter H, Bailey A, Ellis J, Prior P (2001). Natural history of cervical human papillomavirus infection in young women: a longitudinal cohort study. Lancet..

[10] Walboomers JM, Jacobs MV, Manos MM, Bosch FX, Kummer JA, Shah KV (1999). Human papillomavirus is a necessary cause of invasive cervical cancer worldwide. J Pathol.

[11] Ronco G, Giorgi-Rossi P, Carozzi F, Confortini M, Dalla Palma P, Del Mistro A (2010). Efficacy of human papillomavirus testing for the detection of invasive cervical cancers and cervical intraepithelial neoplasia: a randomised controlled trial. Lancet Oncol.

[12] Gupta S, Palmer C, Bik EM, Cardenas JP, Nunez H, Kraal L (2018). Self-sampling for human papillomavirus testing: increased cervical cancer screening participation and incorporation in international screening programs. Front Public Health.

[13] Jentschke M, Chen K, Arbyn M, Hertel B, Noskowicz M, Soergel P (2016). Direct comparison of two vaginal self-sampling devices for the detection of human papillomavirus infections. J Clin Virol.

[14] Gyllensten U, Sanner K, Gustavsson I, Lindell M, Wikstrom I, Wilander E (2011). Short-time repeat high-risk HPV testing by self-sampling for screening of cervical cancer. Br J Cancer.

[15] Lindstrom AK, Hermansson RS, Gustavsson I, Hedlund Lindberg J, Gyllensten U, Olovsson M (2018). Cervical dysplasia in elderly women performing repeated self-sampling for HPV testing. PLoS One.

[16] Arbyn M, Anttila A, Jordan J, Ronco G, Schenck U, Segnan N (2010). European guidelines for quality Assurance in Cervical Cancer Screening. Second edition--summary document. Ann Oncol.

[17] CIE TBoHaW (2016). Cancer incidence in Sweden.

[18] Hermansson RS, Olovsson M, Hoxell E, Lindstrom AK (2018). HPV prevalence and HPV-related dysplasia in elderly women. PLoS One.

[19] Lanner L, Lindstrom AK (2020). Incidence of HPV and HPV related dysplasia in elderly women in Sweden. PLoS One.

[20] Gyllensten U, Lindell M, Gustafsson I, Wilander E (2010). HPV test shows low sensitivity of pap screen in older women. Lancet Oncol.

[21] Graneheim UH, Lindgren BM, Lundman B (2017). Methodological challenges in qualitative content analysis: a discussion paper. Nurse Educ Today.

[22] Nelson EJ, Maynard BR, Loux T, Fatla J, Gordon R, Arnold LD (2017). The acceptability of self-sampled screening for HPV DNA: a systematic review and meta-analysis. Sex Transm Infect.

[23] Quincy BL, Turbow DJ, Dabinett LN (2012). Acceptability of self-collected human papillomavirus specimens as a primary screen for cervical cancer. J Obstet Gynaecol.

[24] Dzuba IG, Diaz EY, Allen B, Leonard YF, Lazcano Ponce EC, Shah KV (2002). The acceptability of self-collected samples for HPV testing vs. the pap test as alternatives in cervical cancer screening. J Womens Health Gend Based Med.

[25] Sultana F, Mullins R, English DR, Simpson JA, Drennan KT, Heley S (2015). Women's experience with home-based self-sampling for human papillomavirus testing. BMC Cancer.

[26] Virtanen A, Nieminen P, Niironen M, Luostarinen T, Anttila A (2014). Self-sampling experiences among non-attendees to cervical screening. Gynecol Oncol.

[27] Nahvijou A, Hadji M, Marnani AB, Tourang F, Bayat N, Weiderpass E (2014). A systematic review of economic aspects of cervical cancer screening strategies worldwide: discrepancy between economic analysis and policymaking. Asian Pac J Cancer Prev.

[28] Virtanen A, Anttila A, Nieminen P (2015). The costs of offering HPV-testing on self-taken samples to non-attendees of cervical screening in Finland. BMC Womens Health.

