# Supplementary figures and images for: Elderly women’s experiences of self-sampling for HPV testing
Source: BMC Cancer. 2020 May 26;20:473. doi: 10.1186/s12885-020-06977-0 (PMC7249375; doi:10.1186/s12885-020-06977-0)

**Supplementary material**

Figure 1. Self-sampling instructions.


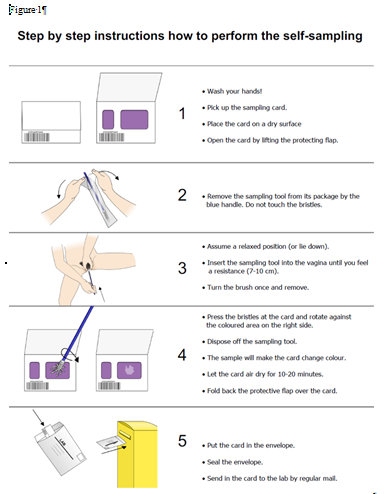

Supplement: Supplementary file 1 — Additional file 1. Fig. 1 Self-sampling instructions. [file 12885_2020_6977_MOESM1_ESM.docx]
